# The Moderating Role of Health Variables on the Association between Physical Exercise and Quality of Life in Patients with End-Stage Renal Disease

**DOI:** 10.3390/healthcare11152148

**Published:** 2023-07-27

**Authors:** Víctor Martínez-Majolero, Belén Urosa, Sonsoles Hernández-Sánchez, David Arroyo

**Affiliations:** 1 Faculty of Human and Social Sciences, Universidad Pontificia Comillas, 28049 Madrid, Spain; burosa@comillas.edu; 2 Faculty of Sports Sciences, Universidad de Castilla la Mancha, 45071 Toledo, Spain; sonsoles@trainsplant.com; 3 Department of Nephrology, Hospital General Universitario Gregorio Marañón, 28007 Madrid, Spain; dvdrry@gmail.com

**Keywords:** physical activity, exercise, quality of life, chronic kidney disease, dialysis, kidney transplant

## Abstract

Scientific evidence demonstrates the positive impact that physical exercise has on the quality of life (QOL) of patients with chronic kidney disease (CKD). However, no study has proposed a model investigating the effect physical exercise has on the QOL of end-stage renal disease (ESRD) patients, considering the most frequent associated diseases (diabetes/hypertension). The objectives were (1) to explore the relationship between physical exercise and the QOL of adults with ESRD, and (2) to examine the moderating and/or mediating role of relevant patient variables. This non-interventional study utilized an ex post facto retrospective data analysis design with a sample of 310 patients with ESRD through two validated questionnaires. The dependent variables were the QOL scale (KDQOL-SF), and the physical function dimension (EFFISICA). The independent variables were the regular practice of intense physical activity (DEPINTE) and the daily time (in hours) the patient is in a sedentary attitude (TiParado). The moderating variables were the clinical situation and associated diseases. The mediator variable used was the body mass index. Bivariate and multiple regression analyses were conducted. Findings suggest implementing intense physical activity in transplant recipients and programmes to avoid sedentary lifestyles in dialysis patients have a positive effect in the QOL of ESRD patients.

## 1. Introduction

The incidence and prevalence of chronic kidney disease is increasing worldwide [1,2,3,4]. Chronic kidney disease (CKD) is one of today’s major public health problems. Worldwide, nearly 500 million people suffer from CKD, and, in Spain, the prevalence is around 15% [5]. This disease has an enormous impact on health-related quality of life (HRQOL) [6]. The scientific literature shows that HRQOL declines as renal function worsens [7,8,9]. The academic literature provides evidence that some of the characteristics associated with better HRQOL in patients with CKD are being a male, married or with a partner, with high level of education, high haemoglobin and haematocrit levels, and kidney transplantation from a living donor. In contrast, female individuals, unemployment, high serum creatinine levels, side effects of immunosuppressive therapy, comorbidities, longer time on dialysis, sedentary lifestyle, and lack of physical activity are negatively associated with HRQOL [6,9,10]. There is empirical evidence demonstrating that resistance exercise improves lower extremity skeletal muscle hypertrophy, muscle strength, and HRQOL in CKD patients in need of renal replacement therapy, but there is limited evidence on the effects of resistance exercise in pre-dialysis CKD patients [11]. In a 2011 study, Maglakelidze et al. compared the HRQOL of a group of patients on haemodialysis, a group on peritoneal dialysis, a group who had rejected their transplant and returned to dialysis, a group of kidney transplant recipients, and a group of healthy individuals of the same age and sex. The results of this study indicated that HRQOL among the haemodialysis and peritoneal dialysis groups were similar and lower than in the healthy population. Renal transplant patients significantly improved their HRQOL to the level of healthy individuals. The failure of a kidney transplant was associated with a significant worsening of HRQOL [12]. Along the same lines, Tomazou et al. (2015) observed that patients on haemodialysis, peritoneal dialysis, and transplantation obtained low scores in quality of life in both the physical health component and general health. These data confirm that kidney transplant recipients obtain better HRQOL scores, followed by peritoneal dialysis patients and, lastly, haemodialysis patients [13]. A fundamental aspect in the difference in the quality of life of these patients is maintaining an active life, which means that treatments that make patients more dependent, such as haemodialysis and peritoneal dialysis, obtain poorer results in quality of life compared to those who are transplanted [14]. In this regard, a study by Dabrowska-Bender et al. (2018) showed that peritoneal dialysis patients scored higher than haemodialysis patients on all dimensions of quality of life. The parameter with the greatest negative impact on haemodialysis patients was the inability to continue working or studying and changes in life plans [15]. In this regard, a review conducted in 2018 showed that kidney transplant patients have a higher rate of life participation (i.e., physical function, travel, recreation, freedom, work) than patients on haemodialysis or peritoneal dialysis [16]. It is necessary to know the HRQOL of patients with CKD to be able to adequately monitor their disease progression and their response to the treatment they are receiving. There are different instruments for measuring HRQOL in these types of patients, and the most widely used instrument is the kidney disease quality of life short form (KDQOL-SF), as it has remarkable psychometric characteristics and has been validated in several languages, including Spanish [17,18].

There are other factors that, according to the scientific literature, are also associated with quality of life in patients with CKD, such as obesity and the development and progression of the disease itself [19]. Body mass index (BMI) is a simple indicator of the relationship between weight and height that is frequently used to identify overweight and obesity in adults, with a score of 25.9–29 being considered overweight, and a BMI > 30 constituting obesity [20]. BMI should be taken with caution, as it does not differentiate between fat mass and lean mass. There are conflicting studies on the association between obesity and mortality. In 2017, Pons Raventos et al. observed that a BMI ≥ 25 kg/m^2^ was not associated with increased mortality, although there was a trend in the data in that direction. Patients with a BMI ≥ 25 kg/m^2^ had a better nutritional status [21]. In contrast, Czyżewski et al., in a 2014, study observed that, together with patient age, increased BMI and hypertension [13] is associated with a drop in HRQOL value in renal transplant and peritoneal dialysis patients [13]. These data suggest that obesity is associated with various structural, haemodynamic, and metabolic alterations in the kidney [22], such as hypertension, hyperglycaemia, inflammation, dyslipidaemia, and atherosclerosis [23]. In addition to obesity, there are other cardiovascular risk factors associated with CKD that are quite common, including age, high blood pressure, cholesterol, diabetes, and a sedentary lifestyle. In a 2018 study, Gorostidi et al. studied the impact of cardiovascular risk factors in Spain and their relationship with the prevalence of CKD, observing that, of the patients diagnosed with CKD, 37.3% were older than 65 years, 61.4% had hypertension, 61.2% had hypercholesterolemia, 16.9% were diabetic, and 72.8% of people were moderately inactive or totally inactive. These data show that certain health problems are associated with CKD and indirectly indicate the importance of being physically active in reducing the likelihood of CKD [5].

Understanding the mechanisms that predispose patients to adaptive renal changes should be directed towards establishing preventive and therapeutic interventions to slow the progression of CKD prevalence [22]. Survival rates correlate with higher levels of exercise and physical function and, consequently, have a benefit in increasing life expectancy in this population [24]. This highlights the importance of carrying out physical exercise programmes, which can be designed by health and physical activity professionals, for chronic kidney patients in their renal replacement therapy [25]. From such findings, it could be derived that interventions focusing on the promotion of physical activity could have a positive influence on the HRQOL of these patients. In fact, there is scientific evidence showing the health benefits of physical exercise, both for the prevention and the treatment of chronic diseases. The presence of physical exercise is associated with a lower incidence of physical diseases, comorbidities, and mortality, as well as a lesser deterioration of psychological and cognitive abilities and a higher HRQOL [26,27].

There is a consensus among nephrologists that physical exercise is important and beneficial for their patients, highlighting the positive effect it has on improving both general health and cardiovascular and aerobic capacity, achieving an improvement in the health of these patients [28,29,30,31,32]. In 2018, Hernández-Sánchez observed that physical exercise programmes in kidney transplant recipients were associated with an improvement in VO2 max, muscle strength, and cardiorespiratory function, with different exercise programmes including a treadmill at home, aerobic exercise performed individually, and exercise programmes supervised by professionals [33]. Similarly, there is evidence of improved VO2 max, physical function, cardiovascular risk factors, muscle atrophy, depression, and quality of life in patients who engage in physical exercise [34,35]. On the same line, physical exercise programmes at home and in the dialysis unit have been shown to be equally effective on physical activity levels, physical function, and sleep quality in haemodialysis patients [36,37]. Despite the health benefits of physical exercise, haemodialysis patients have certain barriers to exercise, such as frequent fatigue, muscle fatigue in the lower extremities, and fear of possible fistula problems [38].

Conversely, physical inactivity and poor exercise capacity in these patients has been associated with poor renal function, impaired psychological and cognitive abilities, and increased risk of death [39,40]. In 2019, Pike et al. demonstrated a positive association between sitting time and greater risk of CKD among patients with a glomerular filtration rate (GFR) ≤30 mL/min/1.73 m^2^. It is speculated that this relationship may be due to reverse causality, as having advanced kidney disease, uraemia, other comorbidities, and consequent fatigue in patients with low GFR may lead to increased sitting time as well as earlier initiation of dialysis [41]. An appropriate dose of physical exercise in CKD patients is a correct, safe, and non-pharmacological treatment, reducing cardiovascular risk and increasing energy metabolism [42]. It could be considered that the prescription of physical exercise might be the key to tackle inactivity, functioning as a therapeutic resource at all ages for health promotion and prevention of chronic diseases [43]. Although there is scientific evidence on the benefits of physical exercise in chronic patients [39], there is no consensus on the specific prescription to be made on the variables of training, indicating the type of exercise, intensity, volume, frequency of exercise, and risk of injury [44]. There is also still insufficient research on the effect and specific interferences that physical exercise would have on cardiovascular risks and CKD with respect to physical exercise and HRQOL.

The prescription of physical exercise for the treatment of CKD has been included in the KDOQI (kidney disease outcomes quality initiative) guidelines since 2001 [45], which recommend three to five aerobic exercise sessions per week with durations varying between 30 and 60 min per session at a low or moderate intensity [46,47]. Following the systematic review of the literature on the treatment of this type of patient by Quiu et al. [4] in 2017, it could be pointed out that both aerobic and strength exercises are recommended. Other studies recommend 30–60 min of moderate exercise 4–7 days per week [48]. Conversely, Gollie et al., in a review in 2018, indicated the importance of monitoring changes in skeletal muscle to know the state of health and progression of CKD. In this study, the recommendations for enduring physical exercise for patients with CKD are very similar to those of the American College of Sport Medicine (ACSM). These are based on 8–10 multi-joint exercises per session performed twice a week. An intensity between 60 and 70% of 1 maximum repetition (RM) or 5 RM is recommended, as well as a gradual increase in volume [11].

An experimental study with a physical exercise programme performed at home or in intradialysis obtained benefits in physical function and physical activity levels in patients. These results were obtained with aerobic resistance and strength exercise, with a load adapted to their 10 RM, with 1–3 sets of 10 repetitions, performed 3 times per week for 16 weeks [36]. In this regard, in 2019, Calella et al. conducted a systematic review on physical exercise in kidney transplant recipients. Due to the great variability in the frequency, duration, and intensity of the exercise programmes of the studies analysed, they were unable to reach conclusions on which exercise is best for this type of patient. However, given that most of the studies repeated the exercise 3 times per week for a period of 3 to 6 months, it could be realistic to suggest an exercise intervention of at least this duration for there to be positive results in this type of patient [49]. Therefore, it is important to encourage patients to be physically active, but the volume and intensity of activity should be tailored to the individual needs of each patient and the severity of CKD [11,50]. In this respect, different approaches can be proposed, such as home exercise programmes, supervised training, or in the hospital’s gym [51].

In view of the above, we understand that physical exercise is important and necessary for a better HRQOL in patients with end-stage renal disease; however, no study has proposed a global model on how and what aspects should be considered for the inclusion of physical exercise in patients with CKD. This study was carried out with the aim of exploring the associations that may exist between the practice of physical exercise and HRQOL, considering other variables that could modify this association. The specific objectives pursued are, firstly, to find out the relationship between physical exercise and quality of life in adults with end-stage CKD and, secondly, to check the moderating and/or mediating role of some relevant variables such as the clinical situation (transplant/dialysis) and associated diseases (diabetes/hypertension) in the analysis of the influence of physical exercise on quality of life in people with end-stage renal disease.

## 2. Materials and Methods

### 2.1. Participants

In this study, a non-probabilistic convenience sampling was carried out using the snowball technique, given the difficulty of obtaining a large clinical sample of this type of patient. To this end, participants were contacted through different platforms: CKD patient associations, events related to this type of disease, and the haemodialysis unit of the Severo Ochoa Hospital in Leganés. The inclusion criteria included not having been admitted to hospital in the four weeks prior to completing the questionnaire used for data collection, to not have a serious clinical situation, psychiatric, or cognitive disorder that would make the patient unable to answer the questionnaire, be of legal age, to have Spanish as their mother tongue, to have end-stage chronic kidney disease (haemodialysis or renal transplant), and to have been on renal replacement therapy for at least three months and stabilised in the treatment.

The study was approved by the ethics committee of the Universidad Pontificia Comillas in the year 2018, as well as by the research ethics committee of the Severo Ochoa Hospital in Leganés in the year 2019. Participation was voluntary. All study participants with chronic kidney disease on renal replacement therapy signed their consent to participate in the study. The collection and processing of the information was carried out in accordance with the privacy and data protection policy of the University, which ensures anonymous treatment and the use of the collected data only for the purposes of this research, as well as the protection of the data and the right of access, rectification, and suppression of the data by the respondent. Informed consent was obtained from all subjects involved in the study.

A total of 310 individuals with end-stage chronic kidney disease with renal replacement therapy, both on dialysis and renal transplant recipients, participated in this study. Specifically, the people with CKD who took part were 151 dialysis patients and 159 kidney transplant recipients. Of these, 182 were men and 128 were women from the 17 autonomous communities of Spain. The sample included individuals between the ages of 21 and 86, with an average age of 51 ± 14 years. Depending on the type of training, 10.3% do not perform specific training, 47.6% perform aerobic endurance, 5.6% perform strength, and 36.5% perform mixed training. The characteristics of the participants are shown in Table 1 and Table 2.

### 2.2. Design and Procedures

The methodology employed in this study is empirical, specifically using an ex post facto retrospective data analysis design. It is a non-interventional clinical study (NIS) The objectives of the study are therefore descriptive and correlational, specifically the analysis of mediation and moderation processes [52].

To conduct the study, several instruments were designed, including a questionnaire for the collection of sociological and health variables and factors, and a second questionnaire, of our own design, on the practice of physical exercise. In the section on physical exercise, the participants of the study were specifically asked about the importance they gave to physical activity, whether they regularly practised intense physical activity (independent variable, DEPINTE), and the time they spent standing in a sedentary attitude (independent variable, TiParado). More specifically, for the first variable, we asked whether the patient regularly practised intense sports/fitness involving a significant acceleration of breathing or heart rate (running, football, cycling, swimming, rollerblading, zumba, aerobics, spinning, weights, crossfit, toning, etc.) for at least 10 consecutive minutes.

The second question focused on the number of hours patients usually spent sitting or lying down at work, at home, commuting, or with friends. Time spent standing (at a desk, sitting with friends, travelling by bus or train, playing cards, or watching TV) is included; however, the time spent sleeping was not incorporated. The questions for the independent variables (DEPINTE, TiParado) were obtained from the physical exercise questionnaire part of the IPAQ questionnaire. In addition, other self-created questions were added to the questionnaire on exercise habits in terms of frequency, type of activity, professional who advises them on training, etc. Specifically regarding the type of exercise, the following question was asked: What training do you do? Aerobic resistance (exercises of a prolonged duration such as running, playing soccer, riding a bike, swimming, rollerblading, zumba, aerobics, spinning, etc.). Strength (exercises that involve overcoming any resistance or weight such as holds, CrossFit, toning, push-ups, sit-ups, squats, etc.). Mixed (aerobic resistance and strength exercises in the same session or in different sessions).

Prior to its application, a validation of the content and structure of the entire survey was carried out through the expert judgement procedure [53]. Four people participated, two experts in research methodology with extensive professional and research experience in the area who technically judged the questionnaire and two renal transplant patients with a history of prolonged CKD who provided information on the understanding of the content of the questions. Specifically, each judge assessed the relevance, adequacy, clarity, and coherence of each item using a 4-point Likert-type scale. All expert ratings were very positive for all items. Where items scored below 3, minor modifications were made to the wording based on suggestions made by experts.

In addition, a second questionnaire focusing on the kidney disease quality of life, short form (KDQOL-SF), was used as the main variable of the study, due to its wide use with this type of patient. This questionnaire was distributed simultaneously with the first one and was chosen due to the specific dimensions it has for assessing the patients’ real conditions, as well as being validated for the Spanish population and having demonstrated a good psychometric characteristics in terms of reproducibility, validity, and sensitivity [17,54].

The instruments that were applied to the participants were structured in two blocks: (1) the self-developed questionnaire, composed of 47 items, collected information on sociodemographic factors (11 items), anthropometric factors (2 items), clinical aspects (16 items), and physical exercise practice before and after CKD diagnosis (18 items), and (2) a block on quality of life included the 36-item kidney disease quality of life short-form (KDQOL-SF) questionnaire, which collects eight dimensions of physical and mental health: physical function (10 items), role limitations due to physical health problems (4 items), role limitations due to emotional health problems (3 items), social function (2 items), psychological well-being (5 items), pain (2 items), vitality/tiredness (4 items), global health perception (5 items), and general health (1 item).

The main dependent variable used was the overall score obtained on the quality of life scale (KDQOL-SF) and the score on the physical function dimension (EFFISICA), as this is the one that reflects the most disabling situation of the subjects. This dimension reflects the limitations that the patient has for carrying out physical activities on a continuum, ranging from the impossibility of doing activities such as dressing and washing oneself without help (0) to carrying out all types of activities including the most strenuous ones without limitations due to their health (100).

The final version of the questionnaire was distributed through the European Commission’s EUSurvey platform to the different CKD patients. This platform was selected because it has the necessary infrastructure to protect the online forms regarding the security and privacy of the data provided by the participants, although the processing of the data was always anonymised.

### 2.3. Statistical Analysis

Statistical analyses were conducted using IBM SPSS statistics version 26 with the data collected from the patients who participated in the study. The confidence level established was 95% (*p* < 0.05).

The analyses were carried out in two phases, the first of which consisted of performing bivariate analyses to verify the existing relationships between the variables collected in the questionnaire (sociological and health factors, as well as the practice of physical exercise) and the quality of life of the patients, and, thus, select the variables that could have a moderating or mediating effect on the relationship between the performance of physical exercise and quality of life. For this purpose, Pearson’s correlation analysis and tests of mean differences using Student’s t-statistics and F-statistics for one-factor analysis of variance were used. The second phase of the analyses consisted of carrying out mediation and moderation analyses using the Macro “Process” Model 10 developed for SPSS by Hayes [55]. Multiple regression analyses were conducted by the bootstrapping method (10,000 samples) to assess the statistical significance of indirect effects [55]. The selection of the variables that formed part of the models were those for which significant associations were found in the phase one of the analyses. The models were structured precisely to be able to explain whether, in certain patient circumstances, physical activity and sedentary time were associated with better outcomes in quality of life in general and in the physical function dimension.

**Figure 1:**
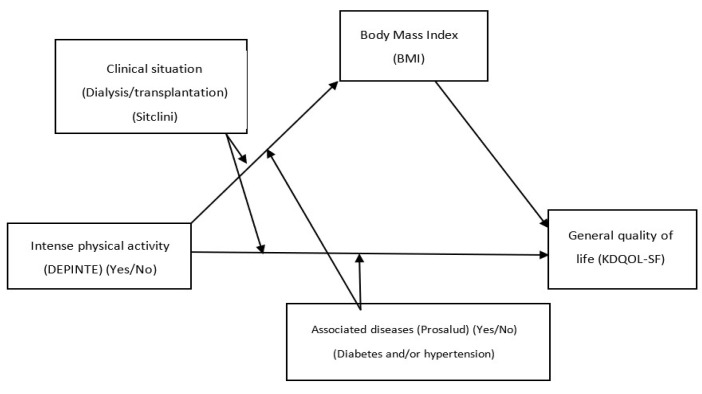
Moderate mediation model of the relationship between the variables of regular practice of intense physical activity (DEPINTE) and general quality of life (KDQOL-SF).

Specifically, two main moderated mediation models were tested. The first (Figure 1) tested the effect of the first independent variable (regularly doing intense sport) on patients’ overall quality of life. Body mass index (BMI) was chosen as the mediating variable, while the moderating variables chosen were clinical status (Sitclini), which identifies whether the patient is transplanted or on dialysis and associated diseases (Prosalud), which identifies patients with a diagnosis of diabetes and/or arterial hypertension. For these analyses, these variables were coded as dummy variables: being on dialysis (−0.50), being transplanted (+0.50), and with associated diseases (−0.50) or without any associated diseases (+0.50).

**Figure 2:**
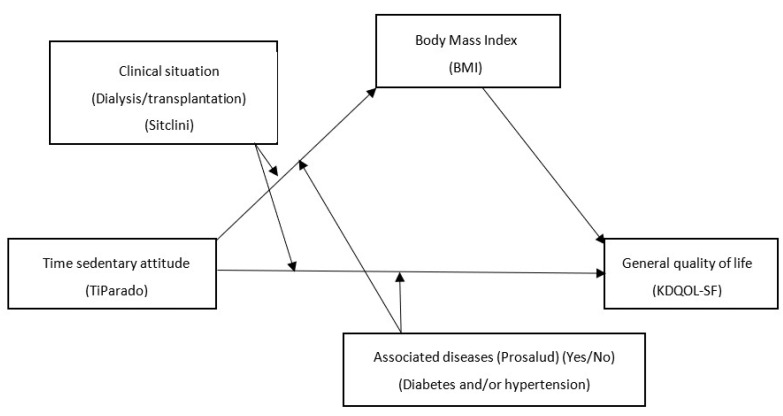
Moderate mediation model of the relationship between the variables time in sedentary attitude (TiParado) and general quality of life (KDQOL-SF).

In the second model (Figure 2), the second independent variable, time in hours per day standing in a sedentary attitude (TiParado), is used as a predictor. The remaining variables acted in the same role as in the first model.

Both models were reiterated using the physical function dimension (EFFISICA) of the KDQOL-SF quality of life scale as the dependent variable.

## 3. Results

The results obtained in the first phase of the analyses were not statistically significant when studying the associations between some of the variables collected on physical exercise habits, such as the frequency with which it is performed, the type of exercising activity carried out, or the professional who advises the training.

However, significant results were found in the association of the main independent variables (regularly practising intense physical activity) and time the patient is standing in sedentary attitude, with the dependent variables of the study: general quality of life and physical function dimension (Table 3).

From the descriptive and bivariate analyses, a relevant result was the importance given by patients to the practice of physical exercise in chronic kidney disease. To this end, they were asked about this importance in the questionnaire through a 0- to 6-point Likert scale. Results showed that both transplant patients (N = 159; M = 5.62; SD = 0.76) and those on dialysis (N = 151; M = 4.97; SD = 1.33) gave high importance to the practice of physical activity, although the group of transplanted patients gave a significantly higher importance than those on dialysis (t = 5.24; *p* < 0.001; d(Cohen) = 0.60).

In the second phase of the analyses, we used the established moderate mediation models with one mediating and two moderating variables.

The results obtained for the model using regular intense physical activity as a predictor variable (Figure 1) are shown in Table 4 for the two dependent variables used in this research. The direct and indirect conditional effects of the model can also be seen in Table 5.

Table 4 shows that being transplanted, having no associated diseases, and engaging in vigorous physical exercise are associated with greater levels of overall quality of life and physical function. For this last variable, a significant interaction was also observed between the clinical situation and the practice of intense physical exercise.

The data in Table 5 show that patients who benefit most in their overall quality of life from regular intense physical exercise are transplant recipients with associated diseases (diabetes/hypertension). Regarding the physical function dimension, transplant patients with associated diseases and dialysis patients, with and without associated diseases (diabetes/hypertension), benefit the most from intense physical exercise.

Results obtained for the second moderated mediation model, which uses the daily time spent standing in a sedentary attitude as a predictor variable (Figure 2), are shown in Table 6 for the two dependent variables used in this research. The direct and indirect conditional effects of the model can also be seen in Table 7.

Table 6 shows that being transplanted, having low BMI levels, and spending more time standing are associated with greater levels of general quality of life and physical function. For the latter variable, a significant interaction between clinical status and time spent out of work was also observed.

Data in Table 7 shows that patients who benefit most in their overall quality of life with less time standing in a sedentary attitude are transplant recipients and dialysis patients with associated diseases (diabetes/hypertension). Regarding the physical function dimension, dialysis patients with and without associated diseases (diabetes/hypertension) benefit the most from less sedentary standing time.

## 4. Discussion

With regard to the first objective of the present study, physical exercise and a less sedentary attitude are associated with a better quality of life in general and in the physical function dimension of quality of life in patients with chronic kidney disease on renal replacement therapy [2,3,4,29,30,31]. However, in this study, clarifications on which physical exercise is the most recommendable for this type of patient could not be made. No statistically significant results were found considering the type and frequency of physical exercise. In the literature, other authors point out that the best results are obtained with mixed training and aerobic resistance training, although, less frequently, there are also positive results with strength training for these patients [49,56]. In the same vein, the dosage of exercise to be performed by these patients is unclear. Above all, the control of training variables, both the type of activity and the intensity, volume and frequency of activity, would be important to achieve the greatest benefits for the body with the lowest risk of injury [44]. These results coincide with those obtained in our study, where the greatest positive relationship with quality of life is not so much based on the type of physical exercise performed, but rather on doing physical exercise regardless of what it is.

In contrast, the results of the study show the importance given to physical exercise by patients with CKD, regardless of whether they perform it or not, with a significant difference between transplant patients and those on dialysis. This leads us to believe that people with this disease know that physical exercise is important, but they do not know how to carry it out in the best possible way and that it does not pose any risk to their health. The clinical situation and health status of dialysis patients could be associated with emotional states that prevent them from seeing physical exercise as a factor that could influence the improvement of their disease. As demonstrated in the scientific literature, differences in exercise practice between transplant and dialysis patients could be due to the low levels of quality of life of dialysis patients, associated with high levels of anxiety and depression [57,58]. These results are in line with other studies, where patients attach importance to physical exercise with a “desire to feel healthy”, but, at the same time, their greatest barrier is lack of motivation [59,60] along with others such as fatigue, changes in health, and the presence of injuries to the musculoskeletal system [61]. Empirical evidence was found proving the importance of being physically active while avoiding a sedentary lifestyle, as more time spent standing has a negative relationship with quality of life, which is in line with other studies in the scientific literature [62,63]. Taking this into account, it is important for the workouts to be individualised and personalised with the intention of achieving greater adherence to them, avoiding injuries, and improving quality of life [47].

Regarding the results provided by the analyses of the moderated mediation models that were carried out in this study, empirical evidence of the direct main effect of the variable practicing intense physical exercise regularly on the general quality of life was found, specifically on the physical function dimension of the patients’ quality of life. Other studies, in which both aerobic resistance and strength training programmes have been implemented for four to six months, also improved the overall quality of life and physical function of these patients [34,35,36,64]. Additionally, this research also verified the effect of the subjects’ clinical situations and having associated diseases on both variables (general quality of life and physical function of quality of life). Transplant patients and patients with no associated diseases have better general quality of life and better physical function. In contrast, dialysis patients have a poorer quality of life. Tomazou et al. (2015), after passing the quality of life questionnaire (KDQOL-SF) to 186 patients with chronic kidney disease, obtained similar results to our study, where transplanted patients obtained better quality of life scores than those on dialysis. However, there are contradictory results as to whether people on peritoneal dialysis have a better quality of life than those on haemodialysis [12,15]. The scientific literature confirms the positive effect of physical exercise on the quality of life of CKD patients, being greater in transplant recipients than in dialysis patients [13,14].

No empirical evidence was found showing the mediating action of the body mass index (BMI) on the relationship between intense physical exercise and quality of life variables. Research by Hyun et al. (2019) demonstrated, through a study with 1880 participants, that waist circumference is a better indicator of quality of life and physical component than BMI, without obtaining a mediating relationship of BMI as the one conducted in this study [65]. The scientific literature identifies BMI as a factor associated with an increased risk of end-stage CKD and a decrease in quality of life [13,66], but there are no studies on its mediating effect. Neither is there a verification of the existence of an interaction between the clinical situation and associated diseases with the intense practice of exercise on general quality of life, which would help indicate in which specific conditions it is more effective. However, it could be concluded that the regular practice of sport and intense physical activity reports better indices of quality of life in general for all the patients analysed.

However, the existence of interaction between the clinical situation and the regular practice of physical exercise was verified, showing that the practice of intense exercise is associated with better indices of quality of life understood as physical function—which allows them to perform any vigorous physical activity—especially in patients who are on dialysis, whether they have associated diseases or not, and in transplanted patients who have associated diseases. In comparison to a review conducted by Chen et al. (2019), physical exercise improves arterial stiffness but does not consistently contribute to the modification of other cardiovascular disease risk factors such as hypertension, dyslipidaemia, hyperglycaemia, decreased renal function, and obesity [67]. This leads us to believe that further studies are needed to understand the specific effect of physical exercise on each cardiovascular risk factor and on the diseases associated with chronic kidney disease.

With respect to the results of the moderate mediation models, in which the predictor variable was the time in sedentary attitude, it was found that, in this case, the body mass index (BMI) does act as a mediator variable in the association with the general quality of life. In other words, being active influences the BMI by reducing it and, through that, improving the general quality of life. BMI is higher in those on dialysis and even greater for those on dialysis and inactive. It is difficult to find in the literature the mediation of BMI on quality of life, although there are studies showing that obesity is associated with changes in the body in renal function, hypertension, hyperglycaemia, dyslipidaemia, and atherosclerosis [22,23]. Therefore, it could be said that the less sedentary the patients are, the more the parameters related to BMI and obesity will be positively modified, and we should expect them to have a higher quality of life. This will have a greater effect on dialysis patients because they are more sedentary due to their changed life plans with less participation in active life [15,16].

No verification of the existence of an interaction between the predictor variable and the moderator variables on the patients’ general quality of life was made. However, statistically significant results were found on the interaction of these variables with the physical function dimension. Being active (not standing) is effective in improving quality of life in terms of physical function, especially in dialysis patients, both for those who have associated diseases and those who do not.

The study has some limitations, as it would have been desirable to select a representative sample of the different patients through randomised procedures. It is also necessary to know the mediating effect taking into account the waist circumference variable, as it is also an indicator of quality of life in these patients. Clinical studies are needed to investigate which type of physical exercise is the most recommendable for this type of population. As a future line of research, it would be interesting to investigate studies with experimental designs that can test the effect of physical exercise with CKD patients on renal replacement therapy and to specifically analyse the type of physical activity, intensity, and frequency that is most recommendable for this type of patient.

## 5. Conclusions

The conclusions drawn from the data provided by this study show that conducting regular intense physical exercise, and not having sedentary attitudes, such as standing still for long periods of time, at a desk at work, sitting with friends, travelling by bus or train, playing cards, or watching TV, have positive benefits in the general quality of life and the physical function dimension of patients with ESRD.

In addition, depending on the clinical situation (transplant recipients or dialysis patients) and associated diseases (diabetes and/or hypertension), the prescription of physical exercise must be different. In transplant patients with associated diseases and on dialysis, whether or not they have associated diseases, the prescription of physical exercise should be focused on performing intense physical exercise on a regular basis and avoiding a sedentary lifestyle, increasing physical activity as far as possible. In addition, these exercise programmes will positively favour the negative effect that have some risk factors, such as age, BMI, hypertension, or diabetes.

## Figures and Tables

**Table 1:** Descriptive data of participants according to clinical situation, gender, associated diseases, dialysis, and previous kidney transplantation.

Variables		N	%
Clinical situation	Renal transplant recipients	159	51.3
	Dialysis	151	48.7
	Total	310	100
Genders	Women	128	41.3
	Men	182	58.7
	Total	310	100
Medical problems	Diabetes	52	16.8
	Hypertension	185	59.7
	Diabetes and hypertension	47	15.1
	Previous dialysis	140	45.2
	Previous transplantation	47	15.2

**Table 2:** Basic descriptive data from participants’ medical records.

Advanced Chronic Kidney Disease	Mean	Standard Deviation
Age (years)	51	13.5
Weight (kg)	72.4	16.1
Height (cm)	166.9	10.8
BMI (kg/m^2^)	26.2	10.1
Time on transplant waiting list (months)	25.5	24.7
Time on dialysis. Patients on dialysis (months)	54.6	77.8
Time duration of previous transplantation (months)	94.1	94.2
Time on dialysis prior to transplant. Transplanted patients (months)	36.1	45.7
Time duration of previous transplant	86.8	87.3
Number of training days per week	3.56	1.51
Exercise time in one session (minutes)	82.06	41.81

**Table 3:** Pearson correlations between the main independent and dependent variables.

	Regular Practice of Intense Physical Activity (Depinte)	Daily Standing Time (TiParado)
General Quality of Life (KDQOLSF)	0.294 ***	−0.284 ***
Physical Function Dimension of Quality of Life (EFFISICA)	0.404 ***	−0.449 ***

*** *p* = 0.000.

**Table 4:** Regression analysis with intense physical activity as predictor variable and clinical status and associated diseases as moderator variables. BMI is a mediator variable, and general quality of life and physical function are dependent variables.

Dependent Variable		B	SE	t	CI (95%)
General Quality of Life (KDQOLSF)	Intense sport (DEPINTE)	4.27	1.69	2.52 *	(0.94; 7.61)
	BMI	−0.13	0.07	−1.92	(−0.27; 0.01)
	Clinical situation (Sitclini)	6.89	1.90	3.62 **	(3.13; 10.64)
Summary of the model: R^2^ = 0.22, F(6, 301) = 13.97 ***	Associated diseases (Prosalud)	−6.39	2.26	−2.82 **	(−10.84; −1.93)
	DepINTExSitclini	2.18	3.11	0.70	(−3.93; 8.31)
	DepINTExProsalud	0.89	3.23	0.28	(−5.47; 7.25)
Physical Function (EFFISICA)	Intense sport (DEPINTE)	16.34	3.29	4.97 ***	(9.87; 22.80)
	BMI	−0.23	0.13	−1.71	(−0.49; 0.04)
Summary of the model: R^2^ = 0.30, F(6, 301) = 21.51 ***	Clinical situation (Sitclini)	20.70	3.70	5.60 ***	(13.43; 27.98)
	Associated diseases (Prosalud)	−12.08	4.39	−2.75 **	(−20.72; −3.43)
	DEPINTExSitclini	−12.64	6.04	−2.09 *	(−24.52; −0.77)
	DEPINTExProsalud	2.15	6.27	0.34	(−10.19; 14.49)

* *p* < 0.05; ** *p* < 0.01; *** *p* < 0.001.

**Table 5:** Direct and indirect conditional effects of intense physical activity as a predictor variable and general quality of life and physical function as dependent variables, with clinical status and associated disease as moderating variables and BMI as a mediating variable.

Dependent Variable		Moderating Variables		Effect	SE	t	CI (95%)
		Clinical Situation (Sitclini)	Associated Diseases (Prosalud)				
General Quality of Life (KDQOLSF)	Conditional direct effect(s) of DEPINTE (DEPINTE > KDQOLSF)	Dialysis (−0.50)	No (−0.50)	2.73	3.42	0.80	(−3.99; 9.46)
		Dialysis (−0.50)	Yes (0.50)	3.63	2.55	1.42	(−1.39; 8.64)
		Transplant (0.50)	No (−0.50)	4.92	2.93	1.68	(−0.84; 10.68)
		Transplant (0.50)	Yes (0.50)	5.81	2.20	2.64 **	(1.48; 10.15)
				Effect	BootSE		BootCI (95%)
	Conditional indirect effects of DEPINTE (DEPINTE > IMC > DQOLSF)	Dialysis (−0.50)	No (−0.50)	0.24	0.31		(−0.28; 0.95)
		Dialysis (−0.50)	Yes (0.50)	0.03	0.28		(−0.30; 0.85)
		Transplant (0.50)	No (−0.50)	0.05	0.24		(−0.27; 0.73)
		Transplant (0.50)	Yes (.50)	−0.16	0.36		(−0.73; 0.70)
				Effect	SE	t	CI (95%)
Dimension Physical Function (EFFISICA)	Conditional direct effect(s) of DEPINTE (DEPINTE > EFFISICA)	Dialysis (−0.50)	No (−0.50)	21.58	6.63	3.26 **	(8.54; 34.62)
		Dialysis (−0.50)	Yes (0.50)	23.73	4.94	4.80 ***	(14.01; 33.46)
		Transplant (0.50)	No (−0.50)	8.94	5.68	1.57	(−2.24; 20.12)
		Transplant (0.50)	Yes (0.50)	11.09	4.28	2.59 **	(2.67; 19.51)
				Effect	BootSE		BootCI (95%)
	Conditional indirect effects of DEPINTE (DEPINTE > IMC > EFFISICA)	Dialysis (−0.50)	No (−0.50)	0.42	0.57		(−0.57; 1.77)
		Dialysis (−0.50)	Yes (0.50)	0.06	0.52		(−0.53; 1.61)
		Transplant (0.50)	No (−0.50)	0.09	0.46		(−0.46; 1.44)
		Transplant (0.50)	Yes (0.50)	−0.28	0.66		(−1.26; 1.41)

** *p* < 0.01, *** *p* < 0.001.

**Table 6:** Regression analysis with time in sedentary attitude as a predictor variable, clinical situation and associated diseases as moderator variables, BMI as a mediator variable, and general quality of life and physical function as dependent variables.

Dependent Variable		B	SE	t	CI (95%)
General Quality of Life (KDQOLSF)	Stopped Time (TiParado)	−0.39	0.21	−1.81	(−0.81; 0.03)
	BMI	−0.14	0.07	−2.09 *	(−0.27; −0.01)
	Clinical situation (Sitclini)	6.79	3.19	2.13 *	(0.51; 13.08)
Summary of the model: (R^2^ = 0.22, F(6, 295) = 14.03 ***	Associated diseases (Prosalud)	−0.11	3.46	−0.03	(−6.92; 6.70)
	TiParado xSitclini	0.03	0.39	0.07	(−0.74; 0.79)
	TiParado xProsalud	−0.89	0.44	−1.88	(−1.68; 0.04)
Physical Function (EFFISICA)	Stopped Time (TiParado)	−1.83	0.40	−4.57 ***	(−2.62; −1.04)
	BMI	−0.23	0.13	−1.81	(−0.49; 0.02)
Summary of the model: (R^2^ = 0.37, F(6, 295) = 28.29 ***	Clinical situation (Sitclini)	−4.53	5.98	−0.76	(−16.29; 7.24)
	associated diseases (Prosalud)	−2.60	6.48	−0.40	(−15.35; 10.16)
	TiParado xSitclini	2.56	0.73	3.52 **	(1.13; 3.99)
	TiParado xProsalud	−1.17	0.82	−1.43	(−2.77; 0.44)

* *p* < 0.05; ** *p* < 0.01; *** *p* < 0.001.

**Table 7:** Conditional direct and indirect effects of time in sedentary attitude as a predictor variable and general quality of life and physical function as dependent variables, with clinical situation and associated diseases as moderating variables and BMI as a mediating variable.

Dependent Variable		Moderating Variables		Effect	SE	t	CI (95%)
		Clinical Situation (Sitclini)	Associated Diseases (Prosalud)				
General Quality of Life (KDQOLSF)	Conditional direct effect(s) of TiParado (TiParado > KDQOLSF)	Dialysis (−0.50)	No (−0.50)	0.01	0.44	0.02	(−0.85; 0.87)
		Dialysis (−0.50)	Yes (0.50)	−0.82	0.24	−3.34 **	(−1.29; −0.33)
		Transplant (0.50)	No (−0.50)	0.04	0.40	0.09	(−0.75; 0.83)
		Transplant (0.50)	Yes (0.50)	−0.79	0.33	−2.35 *	(−1.44; −0.13)
				Effect	BootSE		BootCI (95%)
	Conditional indirect effects of TiParado (TiParado) > IMC > KDQOLSF)	Dialysis (−0.50)	No (−0.50)	−0.06	0.05		(−0.16; 0.05)
		Dialysis (−0.50)	Yes (0.50)	−0.01	0.03		(−0.09; 0.04)
		Transplant (0.50)	No (−0.50)	0.05	0.04		(−0.02; 0.13)
		Transplant (0.50)	Yes (0.50)	0.10	0.08		(−0.02; 0.26)
				Effect	SE	t	CI (95%)
Dimension Physical Function (EFFISICA)	Conditional direct effect(s) of TiParado (TiParado > EFFISICA)	Dialysis (−0.50)	No (−0.50)	−2.53	0.82	−3.07 **	(−4.15; −0.91)
		Dialysis (−0.50)	Yes (0.50)	−3.69	0.45	−8.12 ***	(−4.59; −2.80)
		Transplant (0.50)	No (−0.50)	0.03	0.75	0.04	(−1.45; 1.51)
		Transplant (0.50)	Yes (0.50)	−1.14	0.63	−1.82	(−2.37; 0.09)
				Effect	BootSE		BootCI (95%)
	Conditional indirect effects of TiParado (TiParado > IMC > EFFISICA)	Dialysis (−0.50)	No (−0.50)	−0.09	0.09		(−0.28; 0.70)
		Dialysis (−0.50)	Yes (0.50)	−0.01	0.06		(−0.17; 0.05)
		Transplant (0.50)	No (−0.50)	0.08	0.06		(−0.03; 0.23)
		Transplant (0.50)	Yes (0.50)	0.17	0.13		(−0.04; 0.42)

* *p* < 0.05, ** *p* < 0.01, *** *p* < 0.001.

## Data Availability

Not applicable.
